# Differential Immune Response Associated to Malaria Outcome Is Detectable in Peripheral Blood following *Plasmodium yoelii* Infection in Mice

**DOI:** 10.1371/journal.pone.0085664

**Published:** 2014-01-23

**Authors:** Isabel G. Azcárate, Patricia Marín-García, Alí N. Kamali, Susana Pérez-Benavente, Antonio Puyet, Amalia Diez, José M. Bautista

**Affiliations:** 1 Department of Biochemistry and Molecular Biology IV, Universidad Complutense de Madrid, Facultad de Veterinaria, Ciudad Universitaria, Madrid, Spain; 2 Research Institute Hospital 12 de Octubre, University Hospital 12 de Octubre, Madrid, Spain,; Universidade Federal de Minas Gerais, Brazil

## Abstract

Malaria infection in humans elicits a wide range of immune responses that can be detected in peripheral blood, but we lack detailed long-term follow-up data on the primary and subsequent infections that lead to naturally acquired immunity. Studies on antimalarial immune responses in mice have been based on models yielding homogenous infection profiles. Here, we present a mouse model in which a heterogeneous course of *Plasmodium yoelii* lethal malaria infection is produced in a non-congenic ICR strain to allow comparison among different immunological and clinical outcomes. Three different disease courses were observed ranging from a fatal outcome, either early or late, to a self-resolved infection that conferred long-term immunity against re-infection. Qualitative and quantitative changes produced in leukocyte subpopulations and cytokine profiles detected in peripheral blood during the first week of infection revealed that monocytes, dendritic cells and immature B cells were the main cell subsets present in highly-parasitized mice dying in the first week after infection. Besides, CD4^+^CD25^high^ T cells expanded at an earlier time point in early deceased mice than in surviving mice and expressed higher levels of intracellular Foxp3 protein. In contrast, survivors showed a limited increase of cytokines release and stable circulating innate cells. From the second week of infection, mice that would die or survive showed similar immune profiles, although CD4^+^CD25^high^ T cells number increased earlier in mice with the worst prognosis. In surviving mice the expansion of activated circulating T cell and switched-class B cells with a long-term protective humoral response from the second infection week is remarkable. Our results demonstrate that the follow-up studies of immunological blood parameters during a malaria infection can offer information about the course of the pathological process and the immune response.

## Introduction

The pathophysiological mechanisms that lead to a given outcome in malaria patients are thought to be influenced by epidemiological and immunological factors [1] along with the mechanisms of immune evasion of the parasite [2]. Natural acquired immunity against *Plasmodium falciparum* is incomplete, non sterilizing and can be progressively acquired only after years of repeated infection in adults, but generally not in pregnant women or young children, and does not persist over long periods of time [1].

In the immune response to malaria, innate mechanisms are able to limit parasite density [3], but antibodies (Abs) and T cells are required to completely eliminate blood-stage parasites. APCs are particularly important to activate T CD4 cells which fight against the parasite by producing inflammatory cytokines which activate other cells such as macrophages and helping B cell activation to produce Abs [4]. These Abs have a protective role in malaria [5] and act by blocking merozoite invasion [6], [7], [8], by inhibiting cytoadherence of mature parasite-infected RBCs (iRBCs) [9], by binding to effector cells to trigger parasite-killing effector responses, such as opsonisation and phagocytosis of merozoite or iRBCs [10], [11] or the mechanism known as Ab-dependent cellular inhibition of intracellular parasites [12], [13].

Peripheral blood (PB) sampling has so far been the main provider of information on human immune responses against malaria since it is the only readily accessible source of leukocytes. However, white blood cells (WBC) may not reflect the global response to malaria since the activated cells during the infections may appear in secondary lymphoid organs. Hence, a better understanding of measurable immune system cells and proteins in PB could help identify malaria clinical states in humans. Although studies in animal models have provided useful information on the mechanisms involved in developing protective immunity to malaria, most rodent malaria studies have examined lymphoid organs rather than circulating PB cells because of the large quantity of cells available in these organs. This determines that the extrapolation of experimental data to the human response to infection is not straightforward. A wide variety of host-parasite models have addressed malaria immunity since any single rodent model replicates all the features of human malaria [14]. Despite high genetic variability in human populations, most bioassays in mice have used combinations of *Plasmodium* species and inbred mouse strains, which explains the homogeneous outcomes obtained.

By convention, *P. yoelii yoelii* 17XL (*PyL*) is considered a uniformly lethal parasite strain when used to infect the mouse strains most commonly used, including BALB/c, C57BL/6, Swiss and CBA [15]. Consequently, to date little evidence has been compiled on natural resistance to *PyL* parasites, only DBA/2 strain survives *PyL* infection after developing only moderate parasitemia [16], [17]. Previous results from our laboratory show spontaneous recovery from lethal *PyL* infection of around 20% of the mice from the non-congenic ICR strain [18]. In the present study, we aim to formally characterize this new malaria model and identify potential immune response profiles associated to the different infection courses and final outcome. After a first *PyL* challenge, 20% of outbred ICR mice naturally developed a protective humoral response that confers long-term immunity against homologue re-infections. Besides, repeated individualized cytometric analysis of WBC revealed that cell mobilization and phenotypes vary in mice showing different infection severities and outcomes. Collectively our data revealed dramatic WBC changes that take place during malaria infection and show, for the first time, a heterogeneous course with different blood immune responses to the disease in ICR mice.

## Materials and Methods

### Ethics statement

All animal care and experimental procedures carried out at the Universidad Complutense de Madrid complied with Spanish (R.D. 32/2007) and European Union legislation (2010/63/CE) and were approved by the Animal Experimentation Committee of this institution. The experiments here described involving animals are reported following the ARRIVE guidelines [19]. The number of animals was calculated using Statgraphics Centurion 16.1.18 software (Statpoint Technologies, Inc, Warrenton, VA, USA) to provide about 80% of statistical power with 95% confidence level, and always following the 3Rs principles.

### Animals and parasites

Sixty outbread Hsd:ICR (CD-1) and forty inbred BALB/cAnNHsd pathogen-free female mice, aged 7 weeks were purchased from Harlan Laboratories (Italy) and housed at random in airy racks containing Lignocel® three-fourths bedding (Rettenmaier & Sohne, Rosenberg, Germany) and kept under constant standard conditions of light (12∶12 h light∶dark cycles), temperature (22–24°C) and humidity (around 50%) at the Animal House of the Universidad Complutense de Madrid. All mice were fed a commercial diet (2018 Teklad Global 18% Protein Rodent Diet, Harlan Laboratories) *ad libitum*. The rodent malaria parasite *P. yoelii yoelii* 17XL (*PyL*) was kindly provided by Dr Virgilio Do Rosario (Instituto de Higiene e Medicina Tropical, Universidade Nova de Lisboa) and stored in liquid nitrogen after serial blood passages in mice.

### Experimental infection

Hsd:ICR(CD-1) mice were infected intraperitoneal (i.p.), using a 30 G one-half needle under an approximately 10–15° angle – in the lower quadrant of the abdomen off midline, with 2×10^7^ *PyL*-iRBCs obtained from donor *PyL*-infected mice. Parasitemia was monitored sequentially in each mouse by performing Wright's eosin methylene blue solution-stained thin tail blood smears. Animals that exhibited a loss of weight more than 15% or respiratory distress or other signs of severe disease were sacrificed by cervical dislocation earlier than the final infection endpoint in order to minimize the animal suffering. Total parasite clearance in cured mice was also confirmed by PCR analysis and i.p. sub-inoculation of 50 µl of blood from the mice into naïve BALB/c recipients. Mice that recovered from 1^st^ infection were reinfected on days 60 and 420 post-first infection (pi) following the same infection protocol. RBCs were counted sequentially in each mouse using a hemocytometer and anemia was defined as a RBC number below normal for age (*p*<0.05). Age-matched uninfected mice were used as controls. Three independent experiments are shown (each n = 20).

### Cell preparations

Single-cell suspensions were prepared sequentially from PB, taken from each mouse, for flow cytometry analysis. Around 40 µl of blood were collected from tail of each mouse (without any sign of anxiety or pain during the following 2 hours after the intervention) in PBS containing 0.1 M EDTA and, after RBC lysis with ACK Lysing Buffer (Gibco), WBCs from each animal were individually stained with different mixes of fluorescent Abs. Viable cell counts were always made by Trypan Blue exclusion using a hemocytometer. Total bleeding was always ≤100 µl/mouse/wk to minimize the biological effects of blood loss [20].

### Flow cytometry labeling

Cells from individual mouse were separately incubated with anti-CD16/32 (clone 93; eBioscience) to block non-specific binding, and then stained with different combinations of FITC, PE, PE-Cy5, PerCP-Cy5.5 or APC conjugated anti-CD4 (GK1.5), anti-CD8a (53-6.7), anti-CD45 (30-F11), anti-CD43 (S7), anti-IgM (II/41), anti-CD45R/B220 (RA3-6B2) (from BD Pharmingen); anti-CD44 (IM7), anti-CD5 (53-7.3), anti-CD11b (M1/70), anti-CD23 (B3B4), anti-CD25 (PC61.5), anti-IgD (11-26c), anti-Mac-3 (M3/84), anti-MHC II (M5/114.15.2) (from eBioscience); and anti-CD11c (N418) (from AbD Serotec). Intracellular Foxp3 staining using anti-Foxp3 (FJK-16s) was performed with commercial staining buffer set (eBioscience). Events were acquired on FACSCalibur flow cytometer (BD Biosciences) and data were analyzed with FCS Express software. Adequate isotype controls were used for all Abs (eBioscience). In all tests, cells were firstly gated on a forward scatter-side scatter gate to exclude debris and secondly on a CD45^+^ gate to select the leukocytes. For flow acquisition, >15,000 events in the FSC-SSC gate are collected per sample.

### *P. yoelii* protein extraction from infected whole blood

*PyL* protein lysates were extracted from the whole blood of infected Hsd:ICR(CD-1) mice, which were anesthetized previously by isoflurane inhalation showing >50% parasitemia. Whole blood was collected in tubes containing 0.1 M EDTA and kept at −80°C until protein extraction. The extraction protocol began with erythrocyte lysis using 10 vol of saponin 0.1% (w/v) in PBS. After twice washing in cold PBS, the pellet was treated with 2–3 vol of extraction buffer (50 mM Tris-HCl, pH 8.0; 50 mM NaCl; 0.5% Mega 10; CHAPS 3%) containing a protease inhibitor cocktail (Roche) and subjected to four freeze-thaw cycles. Finally, lysates were centrifuged and *PyL* total protein samples stored at −20°C until use. Protein concentration was determined by the Bradford protein assay (Bio-Rad).

### PCR-quantification of parasite DNA in blood

*P. yoelii* DNA was extracted from peripheral iRBCs using the NuncPrep™ Chemistry Isolation of DNA from Whole Blood protocol of the ABI PRISM® 6100 Nucleic Acid Prepstation (Applied Biosystems) according to the manufacturer's instructions. Oligonucleotide primers and probes for the *P. yoelii yoelii 18S* ribosomal gene subunit (GenBank Accession No. U44379) were taken from [21]. Parasite DNA quantification was assessed employing the 5′ fluorogenic nuclease assay (TaqMan) using a FAMTM dye-labeled specific probe. The primers/probe used were (5′-3′ sequences): Forward, CTTGGCTCCGCCTCGATA; Reverse, TCAAAGTAACGAGAGCCCAATG; Probe, CTGGCCCTTTGAGAGCCCACTGATT. PCR reactions were done in triplicate. Amplification, data acquisition and data analysis were carried out using the ABI 7700 Prism Sequence Detector system (Applied Biosystems).

### Western blotting

10 µg of *PyL* total protein extract were fractionated on 10% SDS-PAGE (Bio-Rad), transferred to nitrocellulose membranes and blocked with 5% non-fat skimmed milk in PBS. Membranes were incubated overnight at 4°C with the different collected sera at 1∶5000 dilutions and then with the secondary HRP-labeled anti-mouse IgG (Amersham Bioscience) at a 1∶5000 dilution.

### ELISAs

Total IgM, IgG and *PyL-*specific IgG Abs were quantified by Ab isotype-specific ELISA. Total IgM and IgG were quantified using anti-mouse IgM or IgG as the capture Ab (Bethyl Laboratories) while *PyL* specific IgG isotypes were quantified using 0.5 µg/well of *PyL* total protein lysates as coating antigen (Ag) prepared in carbonate-bicarbonate buffered solution (Sigma). Coating Ags were incubated for 2 h at RT and subsequently overnight at 4°C. From this step onwards, the manufacturer's protocol was followed (Bethyl Lab.). Briefly, plates were blocked with 1% BSA in Tris-buffered saline solution and duplicate diluted serum samples were added for 1 h at RT (1∶5000 for IgM; 1∶50000 for IgG; 1∶40-1∶2000 for *PyL-*specific IgGs). Total IgM and IgG Abs were detected with HRP-labeled goat anti-mouse IgM or IgG at a 1∶75000 dilution and IgG subclasses were determined with 1∶45000 diluted HRP- labeled goat anti-mouse IgG1, IgG2a, IgG2b or IgG3 Abs. The enzyme reaction was developed using 3,3′,5,5′tetramethyl benzidine (TBM) as the enzyme substrate (Thermo Scientific). Samples were read at 450 nm in a Varian Cary 50 Bio spectrophotometer (Agilent Technologies). Sera from uninfected mice were used as negative controls. Purified myeloma-derived mouse IgG, IgG1, IgG2a, IgG2b, IgG3 and IgM (Bethyl Lab.) were used to generate a logistic four-parameter sigmoidal standard. The lower limit of positivity (cut-off) was determined by the mean of negative healthy controls + 2 SD.

### Adoptive transfer experiments

In groups of 4–5 animals, 6–7 wk-old female BALB/c naïve mice were injected i.v. with 150–200 µg of total IgG from pooled sera obtained from late deceased mice on days 8–11 pi which showed 1.57×10^6^±0.36 ng/ml of total IgGs in serum; or from surviving or uninfected mice on day 70 pi (10 days after the 2^nd^ infection) that had 3.98×10^6^±0.20 ng/ml and 1.70×10^6^±0.72 ng/ml of total IgGs in serum, respectively. Mice injected with PBS were used as infection controls. After 2 h of passive transfer of serum, mice were challenged with 2×10^7^ *PyL* iRBCs. One mouse in each group was left uninfected as a healthy control. Two independent experiments were performed. Animals that exhibited a loss of weight more than 15% or respiratory distress or other signs of severe disease were sacrificed earlier than final infection endpoint in order to minimize the animal suffering.

### Cytokine antibody arrays

To determine cytokines and chemokines in the mouse sera, the Mouse Cytokine Ab Array II kit (AAM-CYT-2-2, RayBio) was used according to the manufacturer's protocol. Pooled serum samples from each group of mice (of days 3 or 7 pi) were applied to the membranes and, after incubation with the detection Ab, membranes were developed with streptavidin-HRP followed by a chemiluminescence reagent (Thermo Scientific). Membranes were then exposed to X-ray film. Pixel densities were calculated for each spot of the array using Quantity One software (Bio-Rad Laboratories) and mean values for duplicate spots were compared. Arbitrary cut-off expression values higher than 1.5-fold were set to consider changes between groups or differences within a group.

### Statistical analysis

To assist sorting of the infected animals into the 3 experimental groups (early deceased, late deceased and surviving), linear regression analysis was used to determine the slope of the parasitemia increase during the first 15 days of the infection. Differences in multiple groups were analyzed using one-way analysis of variance (ANOVA) or Kruskal–Wallis followed, respectively, by the Student's t-test or the Mann-Whitney test to compare significant differences between individual groups in Prism 5 software (GraphPad Software Inc.). Significance was set at *p*<0.05. Data are shown as means ± SEM.

## Results

### Primary *P. yoelii 17XL* infection leads to three malaria infection profiles in ICR mice

**Figure 1:**
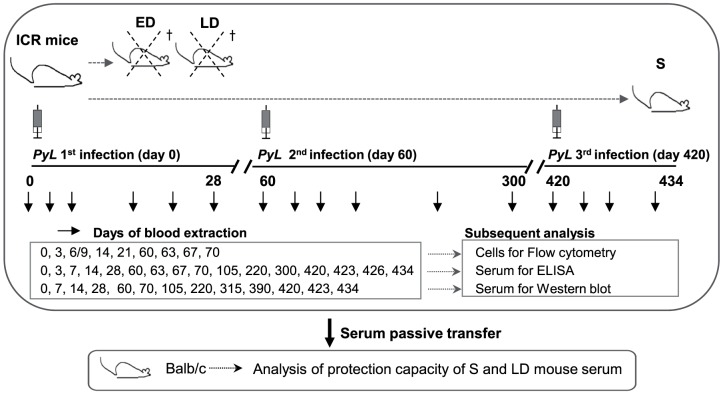
Experimental design used to examine *P. yoelii* 17XL infection in ICR mice. ICR mice were characterized as early deceased (ED), late deceased (LD) or surviving (S) depending on parasitemia rates and outcomes after primary infection with 2×10^7^ *PyL* iRBCs. S mice were reinfected on days 60 and 420 pi. Blood was extracted at the indicated time-points during infection for the different tests using age-matched uninfected mice as controls. The sera of S and LD mice were passively transferred to naïve BALB/c mice.

The i.p. infection with 2×10^7^ *PyL* in ICR outbred mice resulted into three different infection profiles according to their parasitemia and survival kinetics (Table 1). A diagram showing the experimental design is provided in Fig. 1.

**Table 1:** *P. yoelii* 17XL infection in ICR mice leads to three different infection profiles: early death (ED), late death (LD) or survival (S).

	ED mice	LD mice	S mice
% all mice (n = 55)	61.8 (n = 34)	20 (n = 11)	18.2 (n = 10)
Day of death	5.2±0.2 *	11.1±0.6	—
Peak leukocyte number (x10^6^/ml)	21.8±2 *	34.4±7 *	64.1±5.1
Day of leukocyte peak	1.56±0.4 *	6.28±1.6 *	21
Max. iRBCs (%)	83.3±2.1 *	53.7±5	58.8±6.3
Day of max. iRBCs	4.81±0.2 *	8.91±0.6	12±0.7
Max. RBC loss (%)	37.6±6.4	60.2±11	71.8±7.2
Max. RBC loss (10^9^/ml)	3.8± 0.5 * ^S^	4.8±1.3	5.1±1
Day of max. RBC loss	3.2±0.2 *	7.5±1.2	13.4±2.7

*Significant differences between groups (*p*<0.05), except group indicated.

**Figure 2:**
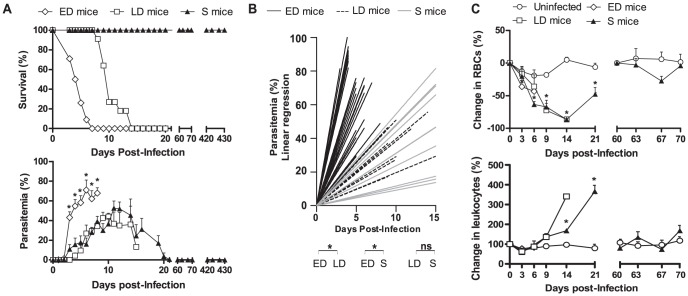
Mice survival and kinetics of parasitemia, anemia and leukocytes in blood of ED, LD and S mice infected with *P. yoelii* 17XL. ICR mice infected with 2×10^7^ *PyL* iRBCs were classified as early deceased (ED), late deceased (LD) or surviving (S) depending on their (*A*) survival and parasitemia. *p<0.05 from ANOVA test. t-test was used to verify that significant differences were only between ED mice and the remaining groups. (*B*) Linear regression of the parasitemia of each mouse. *Significance between the mean 1/slope of each group. (*C*) Changes from baseline produced in circulating numbers of RBCs and WBC. *p<0.05 comparing to uninfected mice. Only one LD mouse was still alive on day 14 pi. Data express mean ± SEM of three independent experiments with n = 20.

A 20% of mice spontaneously resolved the infection and were designated as surviving mice (S). S mice showed a slow increase in parasitemia with a peak of 59% and resolved the infection by day 22 pi (Fig. 2A). The infection was lethal before day 15 pi in the remaining 80% of the animals. Among mice that would die after the infection, two different profiles were observed: Early deceased mice (ED) showed rapid-onset fulminating parasitemia with a peak of 83% and died before day 8 pi (Fig. 2A), being significantly associated the day of their death and the slope of the parasitemia increase (*p*<0.05; R = 0.82). Different from this behavior, the rest of mice which died after infection were designated as late deceased mice (LD) and underwent a slow increase in parasitemia, similar to that of S mice, which peaked at 54%, but followed fatal outcome around day 11 pi. The slope of parasitemia growth was significantly different between ED mice and LD or S mice (both *p*<0.0001), but no differences were found between LD and S mice (Fig. 2B). In LD mice the time of death was not associated with the slope of the parasitemia (*p*>0.05; R^2^ = −0,05). Total clearance of parasites in S mice after infection was confirmed by microscopy examination of blood smears, PCR and sub-inoculation of blood in naïve BALB/c mice. These three infection profiles remained unchanged when the infective dose was 20-fold lower (1×10^6^ *PyL*-iRBCs) (data not shown). To ascertain whether the intrinsic properties of the parasites might contribute to the development of the heterogeneous infection profile observed in ICR, 2×10^7^ *PyL* iRBCs were collected from ICR mice with high-level parasitemia (35%) on day 3 pi and from mice with low-level parasitemia (those with <0.5% on day 3 pi and 35% on day 6 pi) on day 6 to inoculate into 5 BALB/c inbred mice. The course of infection in both BALB/c groups were similar and followed the typical profile since they died on day 6.0±2.3 and day 6.6±2.0, respectively (data not shown).

After their recovery, S mice were reinfected twice on days 60 and 420 pi using the same challenge *PyL* doses. 100% of these animals survived both reinfections and none of them exhibited parasites in PB (Fig. 2A). All mice showed 7.7±0.3×10^9^ RBCs/ml of blood at the beginning of experiment, but anemia was detected in all groups of animals (Fig. 2C). In ED mice, RBC loss was significantly evident from day 3 pi until death (*p* = 0.01) whereas in LD mice, the drop in RBCs started on day 9 pi (*p* = 0.02). In S mice, RBC counts fell from days 6 (*p* = 0.03) to 14 pi (*p* = 0.04), but thereafter recovered and counts were comparable to initial levels. In both LD and S mice, infection induced an increase in WBC (Fig. 2C). In S mice, WBC increased 3.6-fold by day 21 pi (*p* = 0.03), but by the start of the 2^nd^ infection counts returned to baseline.

### Circulating monocytes and DCs show a marked increase in ED mice

**Figure 3:**
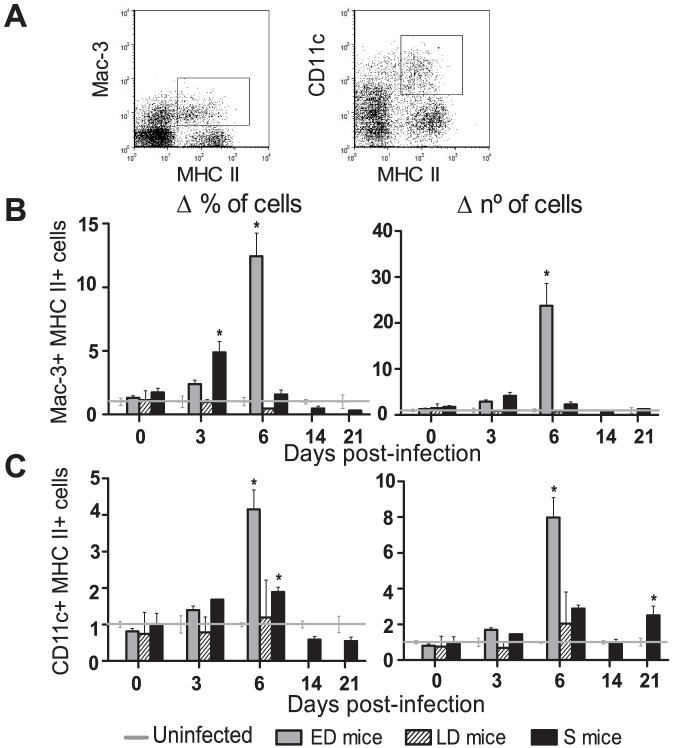
Monocytes and DCs increase in blood of ED mice during acute infection. WBCs were isolated from the PB during the 1^st^ *PyL* infection in ICR mice and (*A*) monocytes (Mac3^+^ MHC II^+^) and DCs (CD11c^+^ MHC II^+^) were detected by flow cytometry. Animals were classified depending on the infection profiles as early deceased (ED), late deceased (LD) or surviving (S) and (*B, C*) their cell frequencies with respect to total leukocytes and numbers were recorded. Data express mean ± SEM of 2 independent experiments, each with n>3 mice per time point. The data for each infected mouse was normalized to the data recorded in 5 uninfected mice per experiment. Initial numbers of macrophages were 10.69±2.57×10^4^ and of DCs were 3.80±0.30×10^5^ per ml of blood.*p<0.05 with respect to uninfected mice.

Both dendritic cells (DCs) [22] and macrophages [23] have been shown to have a protective effect in malaria infection. However, the acute phase of lethal *PyL* may impair DC function [24]. Changes produced in PB activated monocytes (Mac-3^+^MHC II^+^) and DCs (CD11c^+^MHC II^+^) during *PyL* infection and possible links to different outcomes were assessed (Fig. 3A). Initially, mice showed 10.69±2.57×10^4^ macrophages and 3.80±0.30×10^5^ DCs per ml of blood which accounted for a 0.57±0.16% and a 2.05±0.18% of WBCs, respectively. ED mice showed most changes in PB innate immune cells. Thus, 10-fold and 5-fold increases were observed in the frequencies of monocytes and DCs respectively on day 6 pi (Fig. 3B, C; *p*<0.01), when parasitemia was at its maximum level (Fig. 2A). Total cell numbers showed similar kinetics, with an 18-fold increase in monocytes and 10-fold increase in DCs detected on day 6 pi in ED mice (both *p*<0.01). Remarkably, S mice showed the earliest monocyte expansion on day 3 pi (*p* = 0.01) although this increase was reduced relative to the expansion in ED mice on day 6 pi.

### S mice show the enhanced mobilization of CD8 and CD4 T cells

**Figure 4:**
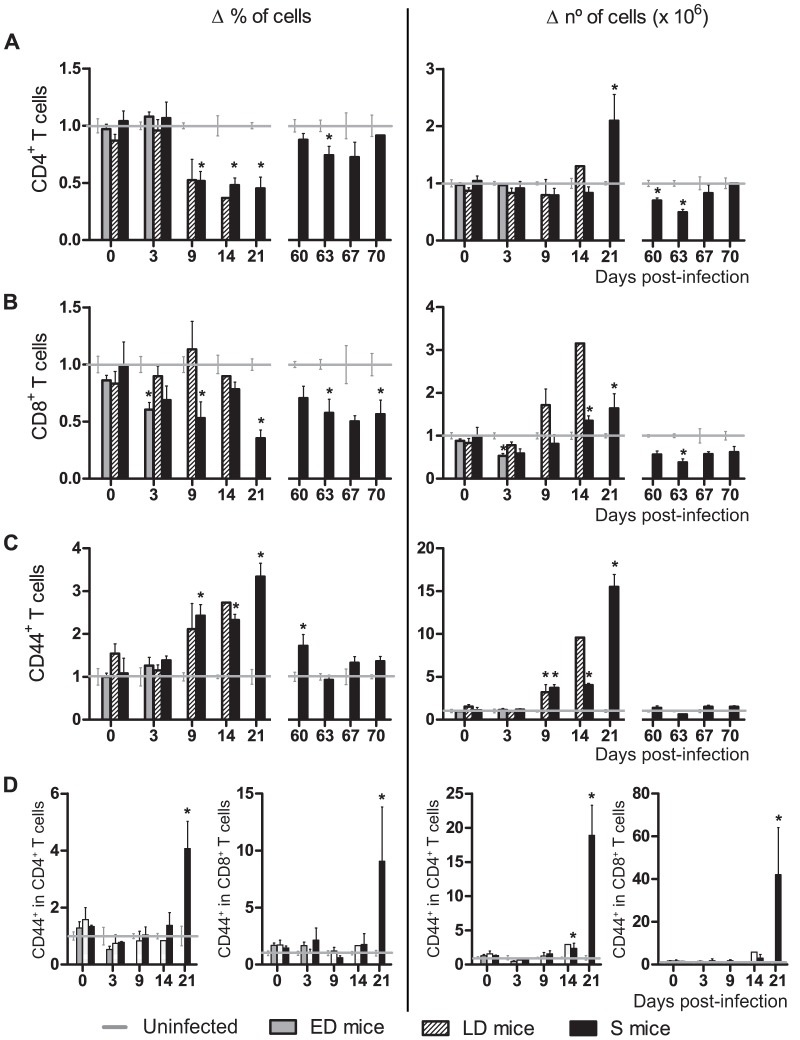
Kinetics of CD4 and CD8 T cells and CD44 expression in blood of *PyL* infected ICR mice. WBCs were isolated from the PB of mice infected with 2×10^7^ *PyL* iRBCs showing different infection profiles: early deceased (ED), late deceased (LD) or surviving (S). Survivors were reinfected on day 60 pi. Frequencies (left panel) and absolute numbers (right panel) of (*A*) CD4 T, (*B*) CD8 T cells, (*C*) total CD44^+^ cells and (*D*) CD44^+^ expressing cells in CD4 and CD8 T populations. Values indicate mean (± SEM) of 2 independent experiments, each with n>3 mice per time point (except day 14 pi with only one LD mouse). The data for each infected mouse was normalized to the data recorded in 5 uninfected mice per experiment. Healthy mice had 4.53±0.12×10^6^ CD4^+^ cells, 1.98±0.04×10^6^ CD8^+^ cells and 2.89±0.19×10^6^ CD44^+^ cells per ml of blood. *p<0.05 with respect to uninfected mice.

It is widely accepted that CD4 T cells are essential to control blood-stage malaria infection and that CD8 T cells play a role during the liver-stage of the parasite cycle [25]. However, the contribution of the latter T cells to blood-stage infection remains unclear. At day 0, mice had 4.53±0.12×10^6^ CD4^+^ cells and 1.98±0.06×10^6^ CD8^+^ cells per ml of blood which constituted a 29.60±0.68% and a 13.10±0.46% of total leukocytes, respectively. In S mice, CD4^+^ and CD8^+^ T cells showed similar kinetics in blood (Fig. 4A, B). Percentages of circulating CD4 and CD8 T cells decreased from day 9 pi onwards during the 1^st^ infection (*p*<0.05, except for CD8 T cells on day 14 pi). In contrast, total numbers of CD4 T cells were elevated on day 21 pi (*p* = 0.01) and numbers of CD8 T cells increased on days 14 and 21 pi (*p* = 0.03). During the 2^nd^ infection in S mice, initial CD4 T cells levels were recovered, but CD8 T cells remained in lower number and proportion than in uninfected mice. ED mice showed a reduction in the number (*p*<0.01) and proportion (*p*<0.01) of CD8 T cells and unchanged CD4 T cell levels, whereas LD mice showed a similar trend to that observed in S mice.

### During primary infection elevated levels of activated lymphocytes are detected in the peripheral blood of surviving mice

CD44 is a ligand for hyaluronic acid, which is up-regulated in activated/memory cells mediating rolling and adhesion during the traffic of activated lymphocytes to target sites of immunity [26]. While standard number and frequency of activated leukocytes (CD44^+^) were 2.89±0.19×10^6^/ml of blood and 18.80±1.10% of total WBCs, these values markedly augmented in S mice, from day 9 to day 21 pi (all *p* = 0.03) (Fig. 4C). To differentiate peripheral T cells, which can be naïve or previously activated Ag-experienced memory cells, we examined their expression of the CD44 receptor. In S mice, the T CD4^+^ population showed a higher frequency of CD44^+^ cells on day 21 pi (*p* = 0.01) and higher CD44^+^ cell numbers on days 14 (*p* = 0.03) and 21 pi (*p* = 0.04). T CD8^+^ CD44^+^ cells showed similar increases on day 21 pi (*p* = 0.03 both frequency and number) (Fig. 4D).

### Circulating CD4^+^CD25^+^ T cell expansion and Foxp3 expression vary with outcome

**Figure 5:**
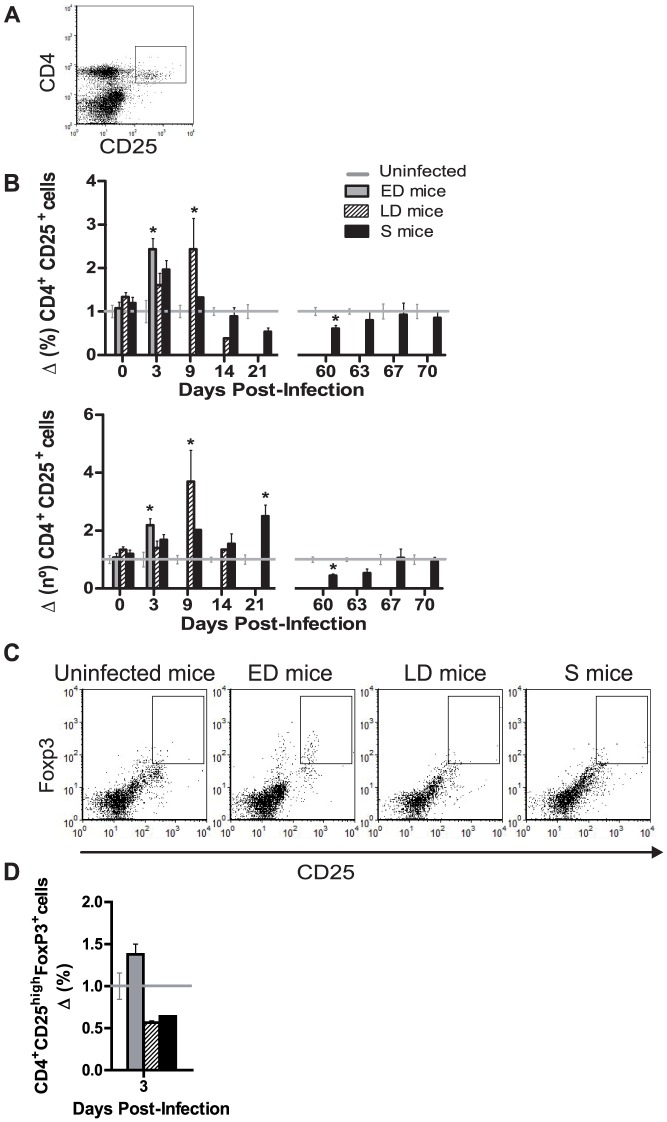
CD4^+^ CD25^+^cells in blood of *PyL* infected mice. ICR mice infected with 2×10^7^ *PyL* iRBCs were classified as early deceased (ED), late deceased (LD) or surviving (S) depending on the infection profiles. Survivors were reinfected on day 60 pi. Blood leukocytes from each mouse were isolated and (*A*) CD4^+^ CD25^+^ cells were detected by flow cytometry. (*B*) Proportions with respect to total leukocytes and absolute numbers recorded in ED mice, LD mice and S mice were normalized to the data recorded in uninfected mice (n = 5 per experiment). Standard values of CD4^+^ CD25^+^ cells were 1.90±0.10×10^5^ cells per ml of blood in healthy mice. * *p*<0.05 with respect to uninfected mice. (*C*) Detection of CD25 and intracellular Foxp3 by flow cytometry gated in CD4^+^ cells. Representative dot plots of the (*D*) frequency of CD4^+^CD25^high^Foxp3^+^ cells at day 3 pi. Standard proportions of CD25^high^Foxp3^+^ cells in CD4^+^ cells were 1,43±0.15%. Data express mean (± SEM) of 2 independent experiments, each with n>3 mice per time point (except day 14 pi with only one LD mouse).

Expression of the IL-2Rα chain, CD25, is a widely used, but not exclusive marker for T regulatory cells (Treg) [27] and the suppressor activity of CD4^+^ CD25^+^ cells is well documented [28], [29] (Fig. 5A). The frequencies and numbers of CD4^+^ CD25^high^ cells were early increased in mice with fatal malaria (Fig. 5B), while in healthy mice numbers remained at levels of 1.90±0.10×10^5^ cells/ml in blood that accounted for a 1.21±0.07% of total WBCs. In S mice, only an increase in cell numbers was detected at the end of the 1^st^ infection (*p* = 0.03) once the parasitemia had been controlled, and these returned to the normal range after the 2^nd^ challenge.

Next, we analyzed the intracellular expression of Foxp3 during the first infection to detect natural Tregs, defined as CD4^+^CD25^high^Foxp3^+^ cells [29] (Fig.5C) and reported to regulate the immune response in malaria [30]. On day 3 pi. the frequency of circulating Tregs increased in ED mice and decreased in LD and S mice (Fig. 5C). Cell levels remained unchanged along the remaining length of the first infection (data not shown).

### Transitional, mature and switched-class B cell kinetics differ according to infection severity

**Figure 6:**
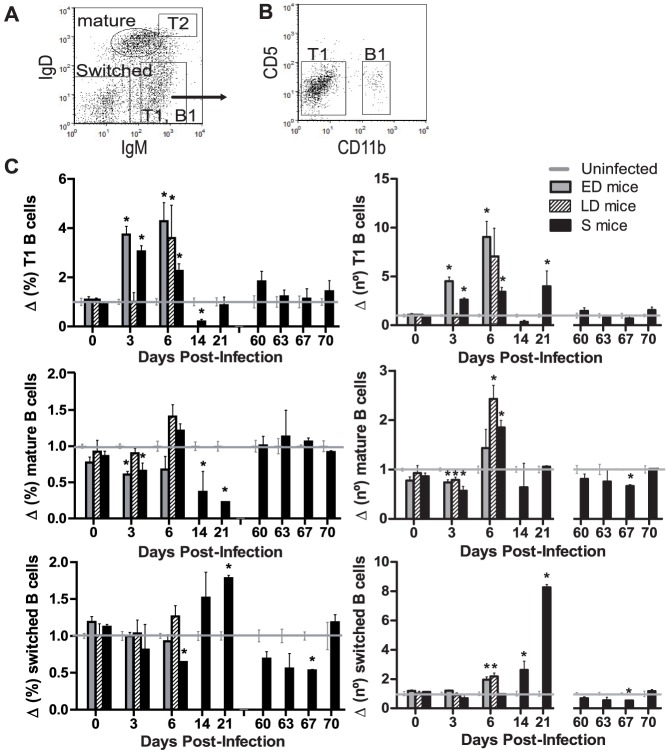
Changes in B cell subpopulations in blood of *PyL* infected ICR mice. ICR mice infected with 2×10^7^ *PyL* iRBCs were classified as early deceased (ED), late deceased (LD) or surviving (S). Survivors were reinfected on day 60 pi. (*A*) Representative flow cytometry dot plot of transitional 1 (T1) and B-1 (IgM^hi^ IgD^low^), transitional 2 (T2) (IgM^hi^ IgD^hi^), mature naïve (IgM^low^ IgD^int^) and class-switched B cells (IgM^−^ IgD^−^) identified among B220^+^ B cells from blood. (*B*) T1 and B-1 cells distinguished through CD5 and CD11b expression. (*C*) Proportions with respect to total B cells (left panel) and absolute numbers (right panel) of B cell subpopulations in mice normalized to the data recorded in uninfected mice (n = 5 per experiment). Data express mean (± SEM) of 2 independent experiments, each with n>3 mice per time point. Healthy mice showed 4.67±0.23×10^6^ of mature B cells and 8.00±0.60×10^5^ of T1 B cells per ml of blood. * *p*<0.05 with respect to uninfected mice.

Mice lacking B cells reveal the importance of B cells in malaria as they are unable to clear *P. yoelii* [31] and *P. chabaudi* infections [32]. To explore the maturation of B cells during blood-stage malaria infection according to severity, the expression of surface IgM and IgD was determined (Fig. 6A). When immature B cells leave the bone marrow, they develop into transitional 1 (T1) (IgM^hi^ IgD^low^) B stage cells, which via the bloodstream reach the spleen. They then become transitional 2 (T2) (IgM^hi^ IgD^hi^) cells and progress to mature cells (IgM^low^ IgD^int^), which re-circulate to find an Ag or remain in the follicular zone of the spleen or progress to marginal zone B cells [33], [34]. After Ag-induced differentiation, B cells commonly switch from expressing IgM to IgG Abs, generating IgM^−^ IgD^−^ cells that include short or long-lived plasma cells and memory B cells [35]. In blood, cells gated as IgM^hi^ IgD^low^ also include B-1 cells [36]. In our study, CD11b^+^ CD5^−^ B-1b cells (B-1a cells, which are CD5^+^, were absent from blood) were detected at very low percentages (always <4.5% of IgM^hi^ IgD^low^ cell levels) while CD11b^−^ CD5^−^ T1 B cells comprised the majority of the IgM^hi^ IgD^low^ cell gate (always >70% of IgM^hi^ IgD^low^ cell levels) (Fig. 6B). All subtypes, except T2 cells, were detected in the PB of all mice. T1 B cells increased their frequency in all infected mice in the first week of infection (*p*<0.05), in contrast to mature and class switched B cell proportions that mainly decreased (*p*<0.01) (Fig. 6C). Healthy mice showed 4.67±0.23×10^6^ of mature B cells and 8.00±0.60×10^5^ of T1 B cells per ml of blood, which accounted respectively for 30.79±1.38% and 5.38±0.40% of total B cells, but the infection produced changes in those values. Although elevated T1 B cell numbers were detected in blood from day 3 in both ED (*p*<0.01) and S mice (*p* = 0.03), the increase was highest in mice with the worst prognosis. Total mature cells presented minor changes; first a decline in all infected mice on day 3 pi and then an increase in LD and S mice on day 6 pi (*p* = 0.03). Isotype-switched B cell numbers showed a small increase in ED (*p* = 0.03) and LD mice (*p*<0.01) on day 6 pi, but in S mice at the end of infection, the increase produced was 8-fold (*p*<0.01).

### The B220^+^ MHC II^+^ subset and proportion of B220^low^ cells

**Figure 7:**
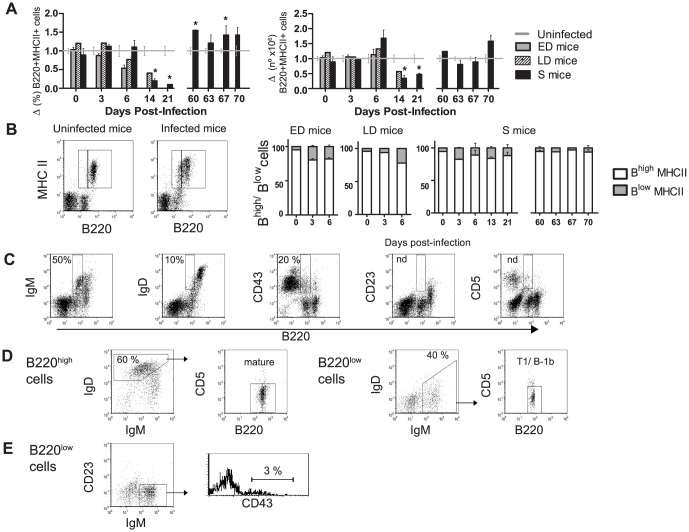
Blood kinetics of MHC II^+^ B cells and their subsets during *P. yoelii 17XL* infection. ICR mice were infected with 2×10^7^ *PyL* iRBCs and classified as early deceased (ED), late deceased (LD) or surviving (S). Survivors were reinfected on day 60 pi. WBCs were isolated and (*A*) B220^+^ MHC II^+^ cells percentages and absolute numbers were normalized to data obtained in uninfected mice (n = 5 per experiment). Standard numbers of B220^+^ MHC II^+^ cells were 4.15±0.17×10^6^/ml blood. * *p*<0.05 with respect to uninfected mice. (*B*) Representative flow cytometry dot plots identifying B220^low^ MHC II^+^ and B220^high^ MHC II^+^ populations in infected mice. Graph shows the contribution of each subpopulation to the total number of B220^+^ MHC II^+^ cells. As uninfected mice (n = 5) maintained in all time-points same proportions as day 0, for simplicity the graphic has not been shown. (*A, B*) Data express mean (± SEM) of 2 independent experiments,each with n>3 mice per time point. (*C*) Dot plot showing IgM, IgD, CD43, CD23 and CD5 expression in B220^low^ cells. (*D*) Identification of mature (IgM^low^ IgD^+^ CD5^−^) and T1 and B-1b cells (IgD^−^ IgM^+^ CD5^−^) in B220^high^ and B220^low^ cell gates. (*E*) In the B220^low^ cell gate, B-1 cells (CD23^−^ IgM^+^ CD43^+^) were distinguished. (*C, D, E*) Representative percentages of measures in 2 independent experiments (n = 10).

MHC class II is constitutively expressed by mature B cells at modest levels, but is dramatically overexpressed on activated B cells and lost after differentiation to plasma cells. Thus, the ability of B cells to up-regulate MHC II expression following activation is likely to be critical for their ability to function as APCs. We examined activated B cells (B220^+^ MHC II^+^) in blood a decrease was observed in their frequency in all infected mice, but only significantly so in S mice on days 14 (*p* = 0.01) and 21 pi (*p*<0.01) (Fig. 7A). Healthy mice showed 4.15±0.17×10^6^ B220^+^ MHC II^+^ cells during all the experiment that was 29.16±1.45% of total WBCs. The absolute B220^+^ MHC II^+^ cell numbers in S mice, even when a gross increase in WBC was observed, represented half the initial cell numbers on day 14 (*p* = 0.02) and day 21 pi (*p* = 0.04). Normal levels of active B cells in PB were recovered at the beginning of the 2^nd^ infection (data not shown).

We could distinguish two populations of B220^+^ MHC II^+^ cells according to the B220 levels: B220^high^ MHC II^+^ (B^high^) and B220^low^ MHC II^+^ (B^low^) as shown in the dot plot in Fig. 7B. Descriptions in the literature already exist of the distribution of B220^high/low^ populations in mice suffering non-lethal *P. yoelii* 17XNL (*PyNL*) infection [37], amyloidosis [38] or mammary tumor virus infection [39] and in healthy neonatal or old mice [40] and ovariectomized mice [41]. Our healthy mice constantly showed about 25% B^high^ cells in PB whereas the B^low^ subset only represented around 1.5% of the total WBC count (data not shown); the normal proportion of B^high^/B^low^ cells being around 9∶1 among total activated B cells (Fig. 7B). However, malaria infection promoted a rise in B^low^ cells and drop in B^high^ cells in all mice from day 3 or 6 pi.

Next, we determined the expression of the different receptors on the B220^low^ cells in all infected mice: all B220^low^ cells were CD23^−^ and CD5^−^, 12.8±2.5% were IgD^+^, 48.2±5.4% were IgM^+^ and 17.8±3% were CD43^+^ (Fig. 7C). Interestingly, the expression of IgM and IgD in B220^low^ and B220^high^ cells in PB revealed that B220^high^ were mainly mature cells (IgM^low^ IgD^+^ CD5^−^) whereas B220^low^ were ∼50% B-1 and T1 B cells (IgD^−^ IgM^+^ CD5^−^) and ∼50% IgD^−^ IgM^−^ CD5^−^ cells (Fig. 7D). Although some studies have defined the B220^low^ population as CD43^−^ B-1b cells [40], classic B-1 cells are IgM^+^ CD23^−^ CD43^+^ IgD^−^ (reviewed in [42], [43]). To examine the presence of B-1 cells in the B220^low^ population, we distinguished CD23^−^ IgM^+^ CD43^−^ T1 cells from classic CD23^−^ IgM^+^ CD43^+^ B-1 cells and only 3.11±0.7% of B-1b cells were detected (Fig. 7E).

### Antibodies in serum during three consecutive *PyL* infections

**Figure 8:**
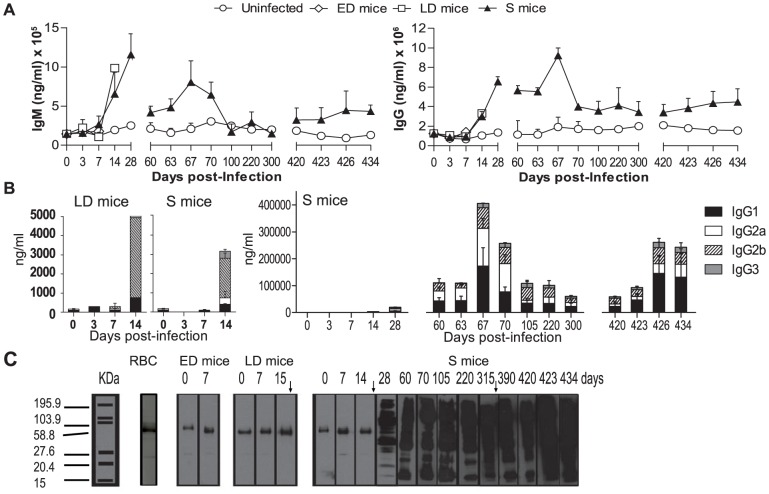
Humoral response following *PyL* infection in ICR mice. ICR mice were infected with 2×10^7^ *PyL* iRBCs and classified as early deceased (ED), late deceased (LD) or surviving (S). Survivors were reinfected on days 60 and 420 pi. (*A*) Total serum IgG and IgM concentrations and (*B*) *PyL*-specific IgG isotypes were analyzed by ELISA during infections. Data express mean (± SEM) of n>5 mouse sera per time point (except day 14 pi, with only one LD mouse). Basal IgG levels against uninfected RBC were similar to that shown on day 0 (data not shown). (*C*) Representative Western blots show the specificity of serum IgGs against uninfected RBC (indicated as RBC) or against *PyL* infected RBCs (indicated as ED, LD and S mice). Western blots were prepared using protein extracts of *PyL* iRBCs and anti-mouse IgG-HRP as secondary Ab.

Abs are crucial components of the protective immune response against malaria in human and animal models [5], [44]. *PyL* infection modifies Ab production in LD and S animals (Fig. 8A). While serum IgM levels peaked in the first infection, IgG Ab production started to increase after the 2^nd^ wk of infection and peaked after the 2^nd^ challenge in S animals. Among the serum *PyL*-specific IgG isotypes, IgG2b were most abundant in S and LD mice (day 14 pi), though IgG2a and IgG1 also reached high levels in S mice (Fig. 8B). In the 1^st^ infection, LD and S mice differed mainly according to the presence of IgG2a in the latter on day 14 pi. The 2^nd^ challenge promoted a rapid 3–4.5-fold expansion in IgG2b, IgG2a and IgG1 and the appearance of IgG3 Abs, which were always the least abundant subclass during all infections. Third *PyL* challenge, induced again the Ab production. To assess specific IgG reactivity, we conducted a time-course IgG immunoblot analysis using total *P. yoelii* proteins (Fig. 8C). IgG Abs recognized a wide range of parasite Ag, the strongest signals appearing in the high molecular weight range. Serum from mice showing a fatal outcome unspecifically reacted with some high molecular weight iRBC proteins. Similar results were obtained using serum from uninfected mice and untreated deceased mice (data not shown). To detect baseline signals, control immunoblots using RBC proteins showed that residual bands recognized by sera from infected mice from day 0 to 14 pi were comparable to those from healthy mice sera and therefore are credited to unspecific recognition of non-infected RBC proteins (Fig. 8C).

### Serum from surviving mice partially protects BALB/c mice from *PyL* infection

To confirm the protective role of anti-*PyL* Abs in S mice, we performed passive transfer assays in BALB/c mice, which is a sensitive strain to *PyL*. Animals were inoculated with 200 µg of serum IgGs taken on day 74 pi from S mice or age-matched uninfected mice or on days 8–11 pi from LD mice and then infected 2 h later with 2×10^7^ *PyL* iRBCs. Transfers using PBS were performed as controls. No protection from infection was conferred by serum from LD mice, uninfected mice or PBS, but pooled sera from S mice was able to cure 40% of transferred naïve mice confirming the protective capacity of anti-*PyL* Abs elicited in spontaneously cured ICR mice (Table 2)

**Table 2:** Passive transfer of serum to BALB/c mice.

Inoculate	Outcome	Mice%	Day of death	Day of max. iRBCs	Max. iRBCs
PBS	Dead	100%	9.3±0.3	8.3±0.3	76±5.5
US	Dead	100%	6.3±1.6	5.7±2	89.7±3
LDS	Dead	100%	6.7±2	6±2.4	91.9±7
SS	Dead	60%	14.7±5.4	13.3±5	81.9±5.3
	Cured	40%	—	15.5±2.1	73.4±0.7

LDS, serum from late deceased mice

SS, serum from surviving mice

US, serum from uninfected mice

Max, maximum

### Circulating cytokines in early deceased and surviving mice

**Figure 9:**
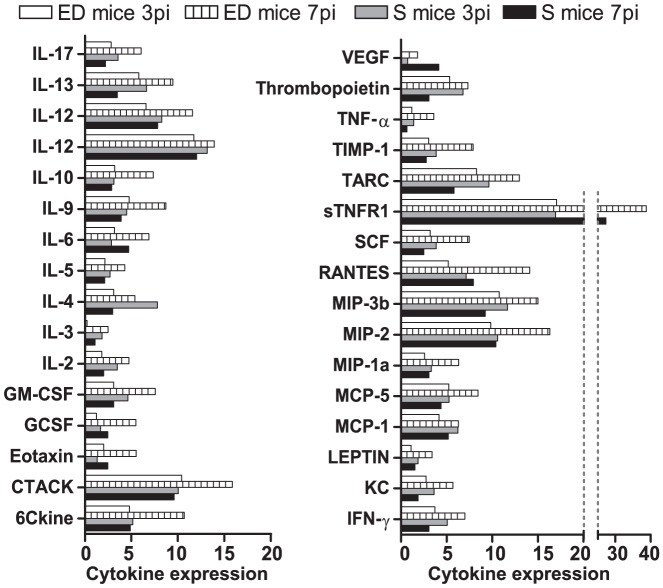
Cytokine antibody array analysis. ICR mice infected with 2×10^7^ *PyL* iRBCs were classified as early deceased (ED) mice or surviving (S) mice. Pooled sera from 4-5 mice in each group collected on days 3 and 7 pi were subjected to the RayBio Mouse Cytokine Array C2 and the density of dots on a membrane for each cytokine was measured and normalized to those obtained using positive Ab array controls.

The balance between pro- and anti-inflammatory responses is essential to limit an immune-mediated disease [45]. In human malaria, evidence exists of a link among cytokine profiles in sera, disease severity and parasitemia [46], [47]. We compared serum cytokine profiles during the 1^st^ wk of infection in S and ED mice by protein microarrays. S and ED mice showed different levels of cytokines examined on days 3 and 7 pi (Fig. 9). At 3 days pi, S mice secreted higher levels of cytokines such as hematopoietic IL-3, the Th2 cytokine IL-4, and the Th1 cytokines IFN-γ and IL-2 than ED mice. Conversely at 7 days pi, ED mice showed higher levels of most of the markers, possibly as the result of their state of terminal decline and consequent physiological dysregulation. At this time point, S mice showed similar cytokine levels in serum to those observed on day 3 pi, though most were overall reduced. Greatest reductions from days 3 to 7 pi were produced in IL-4, TNF-α, IL-13, IL-2, IL-3, IFN-γ and IL-17 while VEGF (vascular endothelial growth factor), eotaxin, IL-6 and sTNFR1 (soluble tumor necrosis factor receptor 1) levels were slightly higher.

## Discussion

Here, we show that the serial analysis of blood immune factors in an animal model yielding different outcomes of infection can provide useful information on the course of the immune response and pathological processes. Our experiments reveal for the first time that the outbred ICR mouse strain mounts different immunobiological responses against primary *PyL* infection associated to the course of infection. Three different infection profiles were observed according to parasitemia and survival: a fulminating parasite growth that led to a fatal outcome before day 8 pi in 60% of animals (designated early deceased, or ED mice) or a more sustained parasitemia that was lethal before day 15 pi in 20% of animals (designated late deceased, or LD mice) yet self-resolved in a further 20% (designated surviving, or S mice). In S mice, parasitemia followed the typical non-lethal *Plasmodium* kinetics for rodents, peaking after 2 wk and clearing by the 3^rd^-4^th^ wk. Although mice surviving a malaria infection usually become immune to following infections, 5% of BALB/c naïve mice that recover from *P. yoelii nigeriensis* primary infection remain susceptible to reinfection a month later [48]. After parasite clearance, homologue reinfections in S mice revealed that these animals develop a protective long-standing immune response for 14 months.

In our model, *PyL* parasite contact promoted a late leukocytosis, consistent with the results of prior murine studies [49], [50], [51], but contrary to reports of acute malaria in humans in which leukocytopenia and lymphopenia are characteristic features [52], [53]. This discrepancy and the drastic changes in mouse blood cell composition observed during blood-stage *P. chabaudi* [49], [50] and *PyL* infections (present study) point to the importance of timing of blood sampling during a malaria infection. Some authors have even recently suggested that immunological studies based on WBC in mice may provide data comparable to the infection process in humans [14].

Our serial cytometric phenotyping of WBC served to identify different cell kinetics in ICR mice according to disease outcome. The first defense barrier against a 1^st^ wave of *Plasmodium* is the innate cell response that promotes the subsequent T cell-mediated response [3]. Macrophages play a critical role in the immune response to malaria due to their ability to phagocytose iRBCs in the absence of cytophilic or opsonizing Abs, to activate T cells through Ag presentation on MHC II and to release inflammatory cytokines. Particularly in *PyL* infections, the key role of macrophages has been clearly demonstrated [23]. The contributions of DCs include Ag uptake and stimulating T-helper cells [54]. In the present study, although ED mice experienced the highest increase in blood levels of both innate cell populations during the 1^st^ wk of infection, the innate response elicited was unable to control the high parasitemia (over 60%). The prompt increase observed here in circulating innate system cells in ED mice indicates that high numbers of circulating monocytes or DCs are not needed to control the infection, rather they could be markers of an inadequate innate response at early time points in acute malaria infection. In S and LD mice, minor changes in circulating innate cells during the first week of infection were observed. Hence, the general “stress condition” caused by severe infection in ED mice probably contributed to the dysregulation of immune responses, also reflected by the over production of the majority of cytokines measured. In human malaria, iRBCs can impair the IFN-γ-induced class II MHC expression on monocytes [55] and hemozoin can inhibit maturation of human monocyte-derived DCs [56], mechanisms that would diminish the T cell responses [55]. However, our data indicate that the MHC II expression and the differentiation of monocytes into DCs expression during murine *Plasmodium* contact *in vivo* are not impaired, as described in other congenic mouse strains [57]. Hence, the low efficiency of monocytes in our ED mice could be the result of an insufficient level of IFN-γ compared to levels in surviving mice, as detected in PB by microarrays at the start of infection. In agreement, higher IFN-γ levels in splenocyte cultures have been reported in resistant DBA/2 than susceptible BALB/c mice during *PyL* infection [58].

Neither T nor B lymphocytes in general seem to be required to control the 1^st^ wave of *P. yoelii* infection [23]. However, the suppressive role of CD4^+^CD25^+^ cells [28], [29] make of this population an important player during bacterial [59], viral [60], helminth [61], [62] or protozoan parasite infections including malarial [63] [60], [61], [62], [63], [64]. In the CD4^+^CD25^+^ population are included effector CD4 T cells and Treg cells which play an important role through suppression of the Th1 response [30]. In our study, the increase of PB CD4^+^CD25^+^ cells observed in mice with a fatal outcome occurred before the parasitemia peak, what could be impairing the development of effective protective immunity [30], [65], whereas in surviving mice it was only observed when parasitemia almost cleared. In our mouse model and other mouse strains [66] expansion of CD4^+^CD25^+^ cells correlates with disease outcome. CD4^+^CD25^high^Foxp3^+^ Treg cells followed different kinetics in PB according to parasitemia. Lower percentages where found in mouse groups with lower parasitemias (LD and S mice), while mice with high parasitemias and lethal outcome after a malaria infection was accompanied by an increase of Tregs at the beginning of infection. Thus, the suppressive activity that Foxp3 expression confers to naive T cells [67], might not be beneficial in the first days of malaria infection according to our results.

In the first week of infection, when most of the mice succumbed to the infection, T1 B cells showed the greatest changes among all the B subpopulations. T1 B cells increased the frequency in all infected mice, in contrast to the rest of B cells, and their numbers were particularly enlarged in ED mice blood. This may suggest diminished competence of the immune response since peripheral immature B cells lead to B cell tolerance in the absence of T cell help [68], [69], [70].

A subset of B cells expressing low levels of B220 protein was observed in infected mice. Although it has been speculated that B220^low^ cells might be beneficial in malaria *PyNL* infections [37], similarly increased PB levels of these cells were observed in all our mouse groups and we characterized them mainly as immature B cells and not as B-1 B cells as previously described [37].

From the second week of infection, a late increase in activated T and class-switched B cells, together with the high production of specific Abs, conferred to S mice a very different immunological profile than in the first week. The role of CD8 T cells showed by transfer experiments in *P. yoelii* malaria models is still controversial [71], [72], [73]. Here, the increase in the total number of CD8^+^ cells with an activated phenotype detected here in S mice could point to a protective role or at least suggest they do not impair a proper immune response.

In S mice, the decline of B cells acting as APCs (B220^+^ MHCII^+^) in blood paralleled to class-switched B cells increase in the PB could suggest the exit of B cells from the circulating population to lymphoid organs upon stimulation by parasite Ag, causing them to switch class to Ag-experienced and memory B cells that lose the expression of MHC II as no longer need to bind or present Ag [74]. The inability of erythrocytes to present Ags prevents iRBC destruction by a specific MHC-restricted T-cell response. Immunity to blood stage malaria parasites is thus primarily conferred by humoral immune responses in which the participation of IL-4 producing CD4 Th2 cells and B cells are of major importance [75], [76]. In S mice, an efficient humoral immune response was mounted during 1^st^ infection and maintained for more than a year after the second infection. Microarray analysis showed that IL-4 increased in S mice serum before than in ED mice, what could induce an effective production of Abs [77]. In LD and S mice the parasitemia course as well as general changes in WBC populations and humoral response in the first weeks were quite similar, suggesting that the death of LD animals could be in part due to ineffective erythropoiesis, as described in both human and rodent malaria infection [78].

The development of immunological memory in the S animal group was patent since both reinfections produced a rapid protective specific Ab response. All the IgG isotypes examined, IgG1, IgG2a, IgG2b and IgG3, were detected in the sera of S mice, consistent with findings in *PyNL*-infected ICR mice [79]. In the first infection, the cytophilic IgG2a and IgG2b Abs [80], that would be induced by a Th1 response [81], [82], predominated over non-cytophilic IgG1 and IgG3 Abs [80] that would respond to Th2 response [81], [82]. However, this prevalence decreased in the following infections suggesting the establishment of a Th1 immune response during the first infection and a Th2 response in the reinfections. Studies in humans suggest that the presence of malaria-specific Abs may be dependent on the presence of chronic parasitemia [83], but the clearance of blood parasitemia in *PyL*-ICR mice after each infection was confirmed by microscopy, PCR and re-inoculating blood in naïve mice. The persistence of parasites in the spleen or other organs was, however, not investigated. Our immunoblots revealed that the repertoire of *PyL* Ag was recognized by the specific IgGs raised after each reinfection. This has also been observed in acquired immunity to human malaria and is likely to depend on the build-up of a wide range of antigenic specificities over a long period [84]. In our malaria model, parasite proteins exhibiting antigenicity spanned a wide MW range. Remarkably, circulating Abs against high MW *PyL* Ags were preferably maintained after several months without parasite re-exposure. In a recent proteomic study, we identified some of these *PyL* Ags with Abs from protected S mice, as a new strategy to develop multi-Ag-based vaccine therapies [85]. The goal of the serum-transfer experiments was to determine the protective capacity of the humoral response at the time reaching its maximum in a given group of infected mice. The passive transfer of hyperimmune sera has shown the concept that immunity to *PyNL* [44], [86] and to lethal *P. yoelii nigeriensis* [48] is largely humoral, and this is now extended to include the *PyL* strain by our passive transfer results.

**Figure 10:**
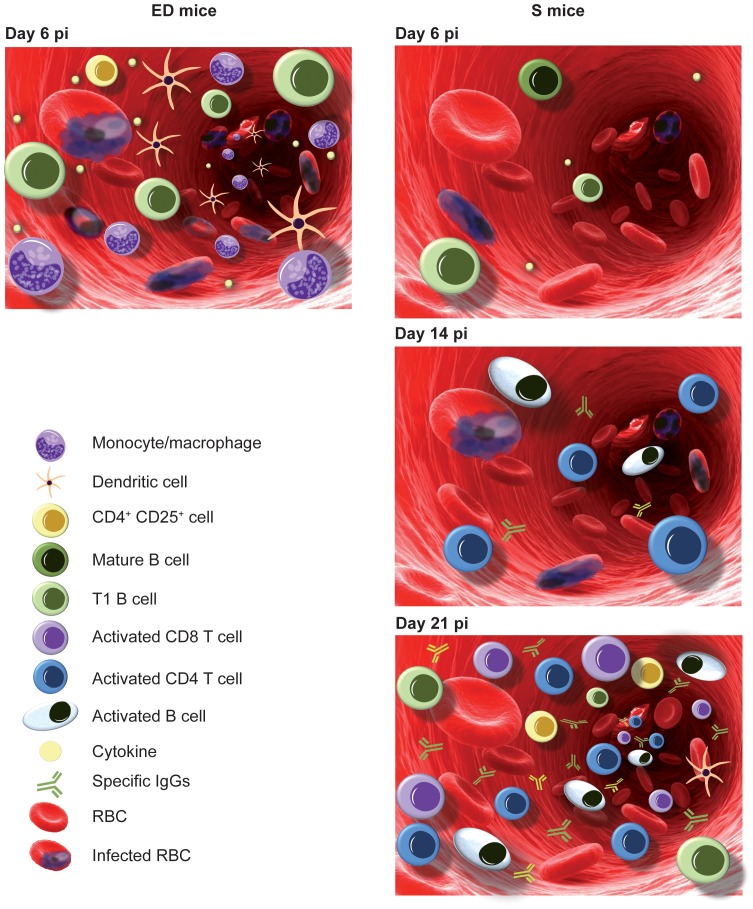
Summary of immune response in peripheral blood of ICR mice with different clinical courses of *PyL* infection. *Left*: ED mice on day 6 pi reached high parasitemia, accompanied by a large increase of CD4^+^CD25^+^ cells, immature T1 B cells, innate response cells an overproduction of cytokines. *Right*: S mice on day 6 pi showed low parasitemia with a slight increase in the circulation of mature and immature B cells and a limited production of cytokines. On day 14 pi, S mice reached around 45% parasitemia and increased the number of activated B and CD4^+^ T cells in addition to high levels of circulating anti-parasite specific IgGs. Parasitemia clearance on day 21 pi was concurrent with the highest leukocytosis level, while increase in specific humoral response continued. In S mice the major increases of leukocyte populations in comparison to healthy mice were in activated CD4^+^ and CD8^+^ T cells, class-switched B cells and, to a lesser extent, T1 B cells and CD4^+^CD25^+^ cells.

The results obtained in our rodent malaria model indicate rapid cell changes in the PB of ICR mice during blood-stage *PyL* malaria related to the severity of the infection and outcome (Fig. 10). During the first week of the infection, the immune response observed in the PB of highly parasitized mice (that had the worst prognosis), consisted of a rapid/higher increase in circulating CD4^+^CD25^+^ T cells with higher expression of Foxp3, in T1 B cells and in activated innate cells in comparison with the rest of mice groups. In contrast, the immune response observed in the same week in S mice, showed a limited production of cytokines and mostly unchanged circulating innate cell kinetics. After the first week of infection S mice were characterized by the circulation of activated T and B cells, together with the generation of a long-term protective humoral response.

Here, we demonstrate that outbred mice strains that show different clinical outcomes are valuable animal models to distinguish between potentially effective and ineffective immune responses to malaria. Thus, once described this model, future experiments are necessary to study in detail the immunological mechanisms of malaria susceptibility and protection in ICR mice. Moreover, whether this effect could also take place in humans deserve to be investigated since can eventually allow to identify WBC markers of severity and outcome of malaria infection, particularly useful in clinical studies to assess new vaccine formulations.

## References

[1] Doolan DL, Dobano C, Baird JK (2009) Acquired immunity to malaria. Clin Microbiol Rev 22: 13–36. 19136431 10.1128/CMR.00025-08 PMC2620631

[2] Hisaeda H, Yasutomo K, Himeno K (2005) Malaria: immune evasion by parasites. Int J Biochem Cell Biol 37: 700–706. 15694829 10.1016/j.biocel.2004.10.009

[3] Stevenson MM, Riley EM (2004) Innate immunity to malaria. Nature Reviews Immunology 4: 169–180. 10.1038/nri1311 15039754

[4] Good MF, Engwerda C (2011) Defying malaria: Arming T cells to halt malaria. Nat Med 17: 49–51. 21217684 10.1038/nm0111-49

[5] Cohen S, McGregor IA, Carrington S (1961) Gamma-globulin and acquired immunity to human malaria. Nature 192: 733–737. 13880318 10.1038/192733a0

[6] Blackman MJ, Heidrich HG, Donachie S, McBride JS, Holder AA (1990) A single fragment of a malaria merozoite surface protein remains on the parasite during red cell invasion and is the target of invasion-inhibiting antibodies. J Exp Med 172: 379–382. 1694225 10.1084/jem.172.1.379 PMC2188181

[7] Miller LH, Aikawa M, Dvorak JA (1975) Malaria (Plasmodium knowlesi) merozoites: immunity and the surface coat. J Immunol 114: 1237–1242. 1117140

[8] Epstein N, Miller LH, Kaushel DC, Udeinya IJ, Rener J, et al (1981) Monoclonal antibodies against a specific surface determinant on malarial (Plasmodium knowlesi) merozoites block erythrocyte invasion. J Immunol 127: 212–217. 6165764

[9] Perlmann P, Troye-Blomberg M (2002) Malaria and the immune system in humans. Chem Immunol 80: 229–242. 12058641 10.1159/000058846

[10] Ferrante A, Kumaratilake L, Rzepczyk CM, Dayer JM (1990) Killing of Plasmodium falciparum by cytokine activated effector cells (neutrophils and macrophages). Immunol Lett 25: 179–187. 2126525 10.1016/0165-2478(90)90112-4

[11] Groux H, Gysin J (1990) Opsonization as an effector mechanism in human protection against asexual blood stages of Plasmodium falciparum: functional role of IgG subclasses. Res Immunol 141: 529–542. 1704637 10.1016/0923-2494(90)90021-p

[12] Bouharoun-Tayoun H, Attanath P, Sabchareon A, Chongsuphajaisiddhi T, Druilhe P (1990) Antibodies that protect humans against Plasmodium falciparum blood stages do not on their own inhibit parasite growth and invasion in vitro, but act in cooperation with monocytes. J Exp Med 172: 1633–1641. 2258697 10.1084/jem.172.6.1633 PMC2188756

[13] Lunel F, Druilhe P (1989) Effector cells involved in nonspecific and antibody-dependent mechanisms directed against Plasmodium falciparum blood stages in vitro. Infect Immun 57: 2043–2049. 2659533 10.1128/iai.57.7.2043-2049.1989 PMC313839

[14] Craig AG, Grau GE, Janse C, Kazura JW, Milner D, et al (2012) The role of animal models for research on severe malaria. PLoS Pathog 8: e1002401. 22319438 10.1371/journal.ppat.1002401 PMC3271056

[15] Li C, Seixas E, Langhorne J (2001) Rodent malarias: the mouse as a model for understanding immune responses and pathology induced by the erythrocytic stages of the parasite. Med Microbiol Immunol 189: 115–126. 11388608 10.1007/s430-001-8017-8

[16] Sanni LA, Fonseca LF, Langhorne J (2002) Mouse models for erythrocytic-stage malaria. Methods Mol Med 72: 57–76. 12125155 10.1385/1-59259-271-6:57

[17] Wang QH, Liu YJ, Liu J, Chen G, Zheng W, et al (2009) Plasmodium yoelii: assessment of production and role of nitric oxide during the early stages of infection in susceptible and resistant mice. Exp Parasitol 121: 268–273. 19109953 10.1016/j.exppara.2008.11.013

[18] Moneriz C, Marin-Garcia P, Bautista JM, Diez A, Puyet A (2011) Parasitostatic effect of maslinic acid. II. Survival increase and immune protection in lethal Plasmodium yoelii-infected mice. Malar J 10: 103. 21518429 10.1186/1475-2875-10-103 PMC3107817

[19] Kilkenny C, Browne W, Cuthill IC, Emerson M, Altman DG (2010) Animal research: reporting in vivo experiments: the ARRIVE guidelines. British journal of pharmacology 160: 1577–1579. 20649561 10.1111/j.1476-5381.2010.00872.x PMC2936830

[20] Weaver JL, Broud DD, McKinnon K, Germolec DR (2002) Serial phenotypic analysis of mouse peripheral blood leukocytes. Toxicol Mech Methods 12: 95–118. 20021196 10.1080/10517230290075341

[21] Witney AA, Doolan DL, Anthony RM, Weiss WR, Hoffman SL, et al (2001) Determining liver stage parasite burden by real time quantitative PCR as a method for evaluating pre-erythrocytic malaria vaccine efficacy. Mol Biochem Parasitol 118: 233–245. 11738713 10.1016/s0166-6851(01)00372-3

[22] Stevenson MM, Ing R, Berretta F, Miu J (2011) Regulating the adaptive immune response to blood-stage malaria: role of dendritic cells and CD4(+)Foxp3(+) regulatory T cells. Int J Biol Sci 7: 1311–1322. 22110383 10.7150/ijbs.7.1311 PMC3221367

[23] Couper KN, Blount DG, Hafalla JC, van Rooijen N, de Souza JB, et al (2007) Macrophage-mediated but gamma interferon-independent innate immune responses control the primary wave of Plasmodium yoelii parasitemia. Infect Immun 75: 5806–5818. 17923512 10.1128/IAI.01005-07 PMC2168355

[24] Wykes MN, Liu XQ, Beattie L, Stanisic DI, Stacey KJ, et al (2007) Plasmodium strain determines dendritic cell function essential for survival from malaria. PLoS Pathog 3: e96. 17616976 10.1371/journal.ppat.0030096 PMC1904473

[25] Doolan DL, Hoffman SL (2000) The complexity of protective immunity against liver-stage malaria. J Immunol 165: 1453–1462. 10903750 10.4049/jimmunol.165.3.1453

[26] Siegelman MH, DeGrendele HC, Estess P (1999) Activation and interaction of CD44 and hyaluronan in immunological systems. J Leukoc Biol 66: 315–321. 10449175 10.1002/jlb.66.2.315

[27] Shevach EM (2002) CD4+ CD25+ suppressor T cells: more questions than answers. Nat Rev Immunol 2: 389–400. 12093005 10.1038/nri821

[28] Thornton AM, Shevach EM (1998) CD4+CD25+ immunoregulatory T cells suppress polyclonal T cell activation in vitro by inhibiting interleukin 2 production. J Exp Med 188: 287–296. 9670041 10.1084/jem.188.2.287 PMC2212461

[29] Belkaid Y, Rouse BT (2005) Natural regulatory T cells in infectious disease. Nat Immunol 6: 353–360. 15785761 10.1038/ni1181

[30] Scholzen A, Minigo G, Plebanski M (2009) Heroes or villains? T regulatory cells in malaria infection. Trends Parasitol 26: 16–25. 19914134 10.1016/j.pt.2009.10.004

[31] Weinbaum FI, Evans CB, Tigelaar RE (1976) Immunity to Plasmodium Berghei yoelii in mice. I. The course of infection in T cell and B cell deficient mice. J Immunol 117: 1999–2005. 136472

[32] von der Weid T, Honarvar N, Langhorne J (1996) Gene-targeted mice lacking B cells are unable to eliminate a blood stage malaria infection. J Immunol 156: 2510–2516. 8786312

[33] Loder F, Mutschler B, Ray RJ, Paige CJ, Sideras P, et al (1999) B cell development in the spleen takes place in discrete steps and is determined by the quality of B cell receptor-derived signals. J Exp Med 190: 75–89. 10429672 10.1084/jem.190.1.75 PMC2195560

[34] Chung JB, Silverman M, Monroe JG (2003) Transitional B cells: step by step towards immune competence. Trends Immunol 24: 343–349. 12810111 10.1016/s1471-4906(03)00119-4

[35] Sagaert X, Sprangers B, De Wolf-Peeters C (2007) The dynamics of the B follicle: understanding the normal counterpart of B-cell-derived malignancies. Leukemia 21: 1378–1386. 17495967 10.1038/sj.leu.2404737

[36] Wardemann H, Boehm T, Dear N, Carsetti R (2002) B-1a B cells that link the innate and adaptive immune responses are lacking in the absence of the spleen. J Exp Med 195: 771–780. 11901202 10.1084/jem.20011140 PMC2193734

[37] Kanda Y, Kawamura H, Matsumoto H, Kobayashi T, Kawamura T, et al (2010) Identification and characterization of autoantibody-producing B220(low) B (B-1) cells appearing in malarial infection. Cell Immunol 263: 49–54. 20231018 10.1016/j.cellimm.2010.02.015

[38] Kawabe S, Abe T, Kawamura H, Gejyo F, Abo T (2004) Generation of B220low B cells and production of autoantibodies in mice with experimental amyloidosis: association of primordial T cells with this phenomenon. Clin Exp Immunol 135: 200–208. 14738446 10.1111/j.1365-2249.2003.02361.x PMC1808931

[39] Ardavin C, Martin P, Ferrero I, Azcoitia I, Anjuere F, et al (1999) B cell response after MMTV infection: extrafollicular plasmablasts represent the main infected population and can transmit viral infection. J Immunol 162: 2538–2545. 10072493

[40] Tachikawa S, Kawamura T, Kawamura H, Kanda Y, Fujii Y, et al (2008) Appearance of B220low autoantibody-producing B-1 cells at neonatal and older stages in mice. Clin Exp Immunol 153: 448–455. 18647322 10.1111/j.1365-2249.2008.03709.x PMC2527364

[41] Masuzawa T, Miyaura C, Onoe Y, Kusano K, Ohta H, et al (1994) Estrogen deficiency stimulates B lymphopoiesis in mouse bone marrow. J Clin Invest 94: 1090–1097. 8083350 10.1172/JCI117424 PMC295170

[42] Baumgarth N (2010) The double life of a B-1 cell: self-reactivity selects for protective effector functions. Nat Rev Immunol 11: 34–46. 21151033 10.1038/nri2901

[43] Berland R, Wortis HH (2002) Origins and functions of B-1 cells with notes on the role of CD5. Annu Rev Immunol 20: 253–300. 11861604 10.1146/annurev.immunol.20.100301.064833

[44] Jayawardena AN, Targett GA, Leuchars E, Davies AJ (1978) The immunological response of CBA mice to P. yoelii. II. The passive transfer of immunity with serum and cells. Immunology 34: 157–165. 342396 PMC1457349

[45] Artavanis-Tsakonas K, Tongren JE, Riley EM (2003) The war between the malaria parasite and the immune system: immunity, immunoregulation and immunopathology. Clin Exp Immunol 133: 145–152. 12869017 10.1046/j.1365-2249.2003.02174.x PMC1808775

[46] Cox-Singh J, Singh B, Daneshvar C, Planche T, Parker-Williams J, et al (2011) Anti-inflammatory cytokines predominate in acute human Plasmodium knowlesi infections. PLoS One 6: e20541. 21687657 10.1371/journal.pone.0020541 PMC3110641

[47] Day NP, Hien TT, Schollaardt T, Loc PP, Chuong LV, et al (1999) The prognostic and pathophysiologic role of pro- and antiinflammatory cytokines in severe malaria. J Infect Dis 180: 1288–1297. 10479160 10.1086/315016

[48] Singh B, Nayak BP, Rao KV, Sharma P (2000) Immune responses mediating survival of naive BALB/c mice experimentally infected with lethal rodent malaria parasite, Plasmodium yoelii nigeriensis. Microbes Infect 2: 473–480. 10865192 10.1016/s1286-4579(00)00321-x

[49] Helmby H, Jonsson G, Troye-Blomberg M (2000) Cellular changes and apoptosis in the spleens and peripheral blood of mice infected with blood-stage Plasmodium chabaudi chabaudi AS. Infect Immun 68: 1485–1490. 10678964 10.1128/iai.68.3.1485-1490.2000 PMC97305

[50] Nduati EW, Ng DH, Ndungu FM, Gardner P, Urban BC, et al (2010) Distinct Kinetics of Memory B-Cell and Plasma-Cell Responses in Peripheral Blood Following a Blood-Stage Plasmodium chabaudi Infection in Mice. PLoS One 5: e15007. 21124900 10.1371/journal.pone.0015007 PMC2990717

[51] Langhorne J, Simon-Haarhaus B (1991) Differential T cell responses to Plasmodium chabaudi chabaudi in peripheral blood and spleens of C57BL/6 mice during infection. J Immunol 146: 2771–2775. 1901884

[52] Worku S, Bjorkman A, Troye-Blomberg M, Jemaneh L, Farnert A, et al (1997) Lymphocyte activation and subset redistribution in the peripheral blood in acute malaria illness: distinct gammadelta+ T cell patterns in Plasmodium falciparum and P. vivax infections. Clin Exp Immunol 108: 34–41. 9097908 10.1046/j.1365-2249.1997.d01-981.x PMC1904634

[53] Lisse IM, Aaby P, Whittle H, Knudsen K (1994) A community study of T lymphocyte subsets and malaria parasitaemia. Trans R Soc Trop Med Hyg 88: 709–710. 7886782 10.1016/0035-9203(94)90242-9

[54] Banchereau J, Steinman RM (1998) Dendritic cells and the control of immunity. Nature 392: 245–252. 9521319 10.1038/32588

[55] Schwarzer E, Alessio M, Ulliers D, Arese P (1998) Phagocytosis of the malarial pigment, hemozoin, impairs expression of major histocompatibility complex class II antigen, CD54, and CD11c in human monocytes. Infect Immun 66: 1601–1606. 9529087 10.1128/iai.66.4.1601-1606.1998 PMC108094

[56] Urban BC, Todryk S (2006) Malaria pigment paralyzes dendritic cells. J Biol 5: 4. 16620370 10.1186/jbiol37 PMC1561485

[57] Luyendyk J, Olivas OR, Ginger LA, Avery AC (2002) Antigen-presenting cell function during Plasmodium yoelii infection. Infect Immun 70: 2941–2949. 12010983 10.1128/IAI.70.6.2941-2949.2002 PMC128011

[58] Chen G, Liu J, Wang QH, Wu Y, Feng H, et al (2009) Effects of CD4(+)CD25(+)Foxp3(+)regulatory T cells on early Plasmodium yoelii 17XL infection in BALB/c mice. Parasitology 136: 1107–1120. 19573259 10.1017/S0031182009990370

[59] Kursar M, Koch M, Mittrucker HW, Nouailles G, Bonhagen K, et al (2007) Cutting Edge: Regulatory T cells prevent efficient clearance of Mycobacterium tuberculosis. J Immunol 178: 2661–2665. 17312107 10.4049/jimmunol.178.5.2661

[60] Kinter AL, Hennessey M, Bell A, Kern S, Lin Y, et al (2004) CD25(+)CD4(+) regulatory T cells from the peripheral blood of asymptomatic HIV-infected individuals regulate CD4(+) and CD8(+) HIV-specific T cell immune responses in vitro and are associated with favorable clinical markers of disease status. J Exp Med 200: 331–343. 15280419 10.1084/jem.20032069 PMC2211981

[61] Taylor MD, LeGoff L, Harris A, Malone E, Allen JE, et al (2005) Removal of regulatory T cell activity reverses hyporesponsiveness and leads to filarial parasite clearance in vivo. J Immunol 174: 4924–4933. 15814720 10.4049/jimmunol.174.8.4924

[62] Finney CA, Taylor MD, Wilson MS, Maizels RM (2007) Expansion and activation of CD4(+)CD25(+) regulatory T cells in Heligmosomoides polygyrus infection. Eur J Immunol 37: 1874–1886. 17563918 10.1002/eji.200636751 PMC2699425

[63] Hisaeda H, Maekawa Y, Iwakawa D, Okada H, Himeno K, et al (2004) Escape of malaria parasites from host immunity requires CD4+ CD25+ regulatory T cells. Nat Med 10: 29–30. 14702631 10.1038/nm975

[64] Belkaid Y, Piccirillo CA, Mendez S, Shevach EM, Sacks DL (2002) CD4+CD25+ regulatory T cells control Leishmania major persistence and immunity. Nature 420: 502–507. 12466842 10.1038/nature01152

[65] Torcia MG, Santarlasci V, Cosmi L, Clemente A, Maggi L, et al (2008) Functional deficit of T regulatory cells in Fulani, an ethnic group with low susceptibility to Plasmodium falciparum malaria. Proc Natl Acad Sci U S A 105: 646–651. 18174328 10.1073/pnas.0709969105 PMC2206590

[66] Wu Y, Wang QH, Zheng L, Feng H, Liu J, et al (2007) Plasmodium yoelii: distinct CD4(+)CD25(+) regulatory T cell responses during the early stages of infection in susceptible and resistant mice. Exp Parasitol 115: 301–304. 17084842 10.1016/j.exppara.2006.09.015

[67] Sakaguchi S, Wing K, Miyara M (2007) Regulatory T cells - a brief history and perspective. Eur J Immunol 37 Suppl 1S116–123. 17972355 10.1002/eji.200737593

[68] Allman DM, Ferguson SE, Cancro MP (1992) Peripheral B cell maturation. I. Immature peripheral B cells in adults are heat-stable antigenhi and exhibit unique signaling characteristics. J Immunol 149: 2533–2540. 1383316

[69] Benschop RJ, Melamed D, Nemazee D, Cambier JC (1999) Distinct signal thresholds for the unique antigen receptor-linked gene expression programs in mature and immature B cells. J Exp Med 190: 749–756. 10499913 10.1084/jem.190.6.749 PMC2195635

[70] Sandel PC, Monroe JG (1999) Negative selection of immature B cells by receptor editing or deletion is determined by site of antigen encounter. Immunity 10: 289–299. 10204485 10.1016/s1074-7613(00)80029-1

[71] Imai T, Shen J, Chou B, Duan X, Tu L, et al (2010) Involvement of CD8+ T cells in protective immunity against murine blood-stage infection with Plasmodium yoelii 17XL strain. Eur J Immunol 40: 1053–1061. 20101613 10.1002/eji.200939525

[72] Chandele A, Mukerjee P, Das G, Ahmed R, Chauhan VS (2010) Phenotypic and functional profiling of malaria-induced CD8 and CD4 T cells during blood-stage infection with Plasmodium yoelii. Immunology 132: 273–286. 21039472 10.1111/j.1365-2567.2010.03363.x PMC3050450

[73] Vinetz JM, Kumar S, Good MF, Fowlkes BJ, Berzofsky JA, et al (1990) Adoptive transfer of CD8+ T cells from immune animals does not transfer immunity to blood stage Plasmodium yoelii malaria. J Immunol 144: 1069–1074. 1967271

[74] Calame KL, Lin KI, Tunyaplin C (2003) Regulatory mechanisms that determine the development and function of plasma cells. Annu Rev Immunol 21: 205–230. 12524387 10.1146/annurev.immunol.21.120601.141138

[75] Petritus PM, Burns JM Jr (2008) Suppression of lethal Plasmodium yoelii malaria following protective immunization requires antibody-, IL-4-, and IFN-gamma-dependent responses induced by vaccination and/or challenge infection. J Immunol 180: 444–453. 18097046 10.4049/jimmunol.180.1.444

[76] Taylor-Robinson AW, Phillips RS (1998) Infective dose modulates the balance between Th1- and Th2-regulated immune responses during blood-stage malaria infection. Scand J Immunol 48: 527–534. 9822263 10.1046/j.1365-3083.1998.00437.x

[77] Stevenson MM, Tam MF (1993) Differential induction of helper T cell subsets during blood-stage Plasmodium chabaudi AS infection in resistant and susceptible mice. Clin Exp Immunol 92: 77–83. 8096804 10.1111/j.1365-2249.1993.tb05951.x PMC1554870

[78] Lamikanra AA, Brown D, Potocnik A, Casals-Pascual C, Langhorne J, et al (2007) Malarial anemia: of mice and men. Blood 110: 18–28. 17341664 10.1182/blood-2006-09-018069

[79] White WI, Evans CB, Taylor DW (1991) Antimalarial antibodies of the immunoglobulin G2a isotype modulate parasitemias in mice infected with Plasmodium yoelii. Infect Immun 59: 3547–3554. 1894361 10.1128/iai.59.10.3547-3554.1991 PMC258919

[80] Grey HM, Hirst JW, Cohn M (1971) A new mouse immunoglobulin: IgG3. J Exp Med 133: 289–304. 5133863 10.1084/jem.133.2.289 PMC2138907

[81] Abbas AK, Murphy KM, Sher A (1996) Functional diversity of helper T lymphocytes. Nature 383: 787–793. 8893001 10.1038/383787a0

[82] Stevens TL, Bossie A, Sanders VM, Fernandez-Botran R, Coffman RL, et al (1988) Regulation of antibody isotype secretion by subsets of antigen-specific helper T cells. Nature 334: 255–258. 2456466 10.1038/334255a0

[83] Akpogheneta OJ, Duah NO, Tetteh KK, Dunyo S, Lanar DE, et al (2008) Duration of naturally acquired antibody responses to blood-stage Plasmodium falciparum is age dependent and antigen specific. Infect Immun 76: 1748–1755. 18212081 10.1128/IAI.01333-07 PMC2292892

[84] Kinyanjui SM, Mwangi T, Bull PC, Newbold CI, Marsh K (2004) Protection against clinical malaria by heterologous immunoglobulin G antibodies against malaria-infected erythrocyte variant surface antigens requires interaction with asymptomatic infections. The Journal of infectious diseases 190: 1527–1533. 15478055 10.1086/424675

[85] Kamali AN, Marin-Garcia P, Azcarate IG, Diez A, Puyet A, et al (2012) Plasmodium yoelii blood-stage antigens newly identified by immunoaffinity using purified IgG antibodies from malaria-resistant mice. Immunobiology 217: 823–830. 22658767 10.1016/j.imbio.2012.05.002

[86] Freeman RR, Parish CR (1981) Plasmodium yoelii: antibody and the maintenance of immunity in BALB/c mice. Exp Parasitol 52: 18–24. 7238723 10.1016/0014-4894(81)90056-4

